# Pseudocyst of the Pancreas Masquerading as Adrenal Myelolipoma: Lessons Learned

**DOI:** 10.7759/cureus.88828

**Published:** 2025-07-26

**Authors:** Thalapathi Raja, Sushmitha Kothapalli, Velmurugan Palaniyandi, Hariharasudhan Sekar, Sriram Krishnamoorthy

**Affiliations:** 1 Urology, Sri Ramachandra Institute of Higher Education and Research, Chennai, IND; 2 Urology, Sri Ramachandra Institute of Higher Education and Research, chennai, IND

**Keywords:** adrenal mass, pancreas pseudocyst, pancreatic fistula, pancreatic leak, pancreatic pseudocyst

## Abstract

A 56-year-old female presented with left loin pain, intermittent vomiting, and generalized weakness, alongside newly diagnosed diabetes mellitus and hypertension. Initial evaluation revealed a firm, vague mass in the left hypochondrium. Contrast-enhanced magnetic resonance imaging and computed tomography of the kidney, ureter, and bladder showed features consistent with left adrenal myelolipoma, promoting laparoscopic adrenal surgery. During surgery, it was revealed that the mass was adhered to the spleen, tail of the pancreas, and left kidney, and hence laparoscopy was converted to open surgery. In the postoperative period, the patient had a foul-smelling discharge, suggesting a pancreatic duct leak. Therefore, the patient was taken up for emergency splenectomy, distal pancreatectomy, removal of the splenic fissure, and transverse colostomy. Histopathological examination revealed a cystic mass, chronic pancreatitis, and inflammation, with no signs of malignancy. The clinical, investigative, and surgical findings were not indicative of an adrenal mass, emphasizing the need for a team approach and careful assessment while diagnosing retroperitoneal problems.

## Introduction

As a result of acute/chronic pancreatitis, fluid-filled spaces are formed around the pancreas. These spaces are known as pancreatic pseudocysts [1]. These cysts can spread to various regions of the abdomen, including the omentum, mediastinum, lesser sac, and groin [2]. In some instances, these might mimic other abdominal masses, making the diagnosis difficult [3].

A pancreatic pseudocyst may mimic an adrenal myelolipoma. Adrenal myelolipoma is a rare benign tumor consisting of mature fat and smooth muscle. Pancreatic pseudocysts and adrenal myelolipoma have similar symptoms, such as nausea, vomiting, and abdominal pain. Hence, rather than relying on clinical symptoms, imaging studies can help accurately diagnose pancreatic pseudocysts. They can help in differentiating pseudocysts from other abdominal masses, including adrenal myelolipomas, and, thus, proper treatment can be administered [4-5].

A study conducted at St. George’s Hospital in London among 40 patients with pancreatic pseudocysts showed that the main causes of pancreatic pseudocysts are acute pancreatitis, chronic pancreatitis, or trauma [6]. The majority of the patients had a pseudocyst in the lesser sac; it was also seen between the layers of the omentum or in the chest or neck. This accentuates how a pancreatic pseudocyst may imitate an adrenal myelolipoma, thereby making a proper diagnosis difficult. Thus, thorough clinical examination, imaging, and histopathological investigation are imperative for accurate diagnosis.

## Case presentation

A 56-year-old female presented to the outpatient department (OPD) with complaints of left-sided abdominal pain persisting for seven days. The pain was associated with intermittent vomiting, low-grade fever, and fatigue over the preceding month. Her medical history was significant for type II diabetes mellitus and hypertension. She was on oral hypoglycemic agents (metformin 500 mg twice daily) and antihypertensives (amlodipine 5 mg once daily). She had previously undergone surgery for uterine fibroids 14 years ago.

A contrast-enhanced computed tomography (CECT) scan performed at an external facility revealed a well-defined, heterogeneous lesion in the left adrenal region, measuring approximately 6.5 × 10 × 5.0 cm. The lesion demonstrated areas of fat attenuation interspersed with soft tissue density, suggestive of an adrenal myelolipoma. No evidence of calcification or invasion into surrounding structures was noted. Based on these findings, a provisional diagnosis of left adrenal myelolipoma was made, and the patient was referred for further evaluation and management.

On admission, she was conscious, oriented, and hemodynamically stable, with a pulse rate of 85/min, blood pressure of 150/100 mmHg, and oxygen saturation of 100% on room air. Systemic examination revealed no significant abnormalities. Abdominal examination showed all quadrants moving with respiration. The umbilicus was in the midline, and there were no sinuses or scars. A mild fullness was noted in the left hypochondrial region. On palpation, the abdomen was soft and non-tender, with a firm, vague mass palpable in the left hypochondrium extending 3 cm below the left costal margin. The mass had an ill-defined lower border, a smooth surface, and an upper border that could not be delineated. Percussion revealed a dull note over the area, and auscultation did not reveal a bruit.

**Figure 1:**
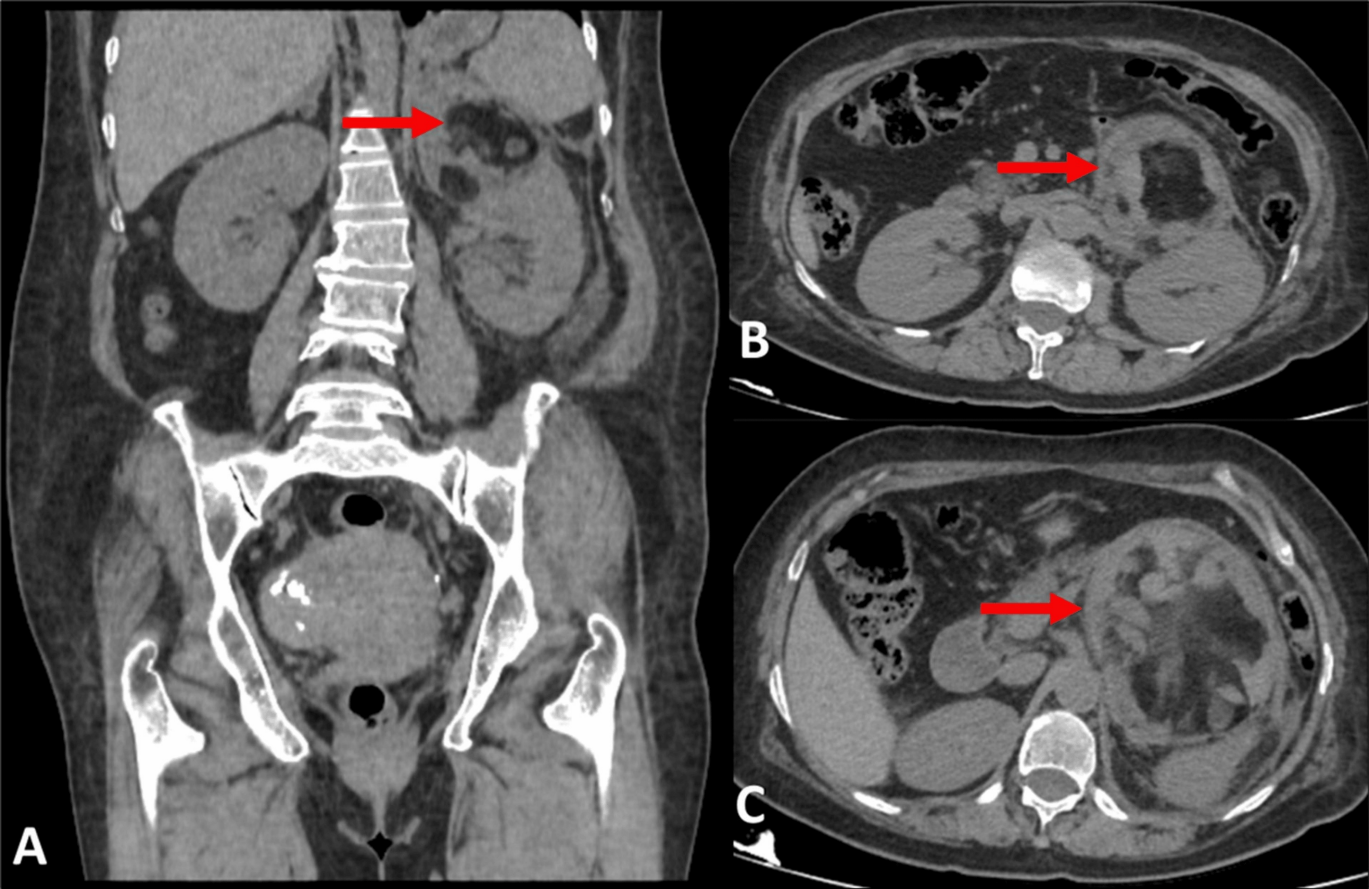
Non-contrast-enhanced computed tomography images. (A) Coronal section showing a retroperitoneal mass in the anterosuperior aspect of the left kidney. (B) Axial section showing the same retroperitoneal mass. (C) Axial section showing the mass abutting the tail of the pancreas.

A CT of the kidney, ureter, bladder confirmed a large, retroperitoneal, heterogeneous lesion with fat attenuation areas (-45 Hounsfield units) and interspersed hyperdense areas, abutting and displacing the tail of the pancreas anteriorly and indenting the superior aspect of the left renal cortex. The left adrenal gland was not visualized. These findings were consistent with a left adrenal myelolipoma (Figure 1). The surgical plan was a laparoscopic left adrenalectomy.

During surgery, the mass was found to be densely adhered to the spleen, splenic flexure, tail of the pancreas, and upper pole of the left kidney, necessitating conversion to open surgery. Adhesiolysis and excision of the mass were performed. Postoperatively, the patient’s drain output remained persistently high, and on postoperative day 4, drain fluid analysis revealed an amylase level >5,000 IU, suggesting a pancreatic duct fistula. Surgical gastroenterology input was sought, and management was adjusted accordingly. The patient was discharged with a drain tube in place.

**Figure 2:**
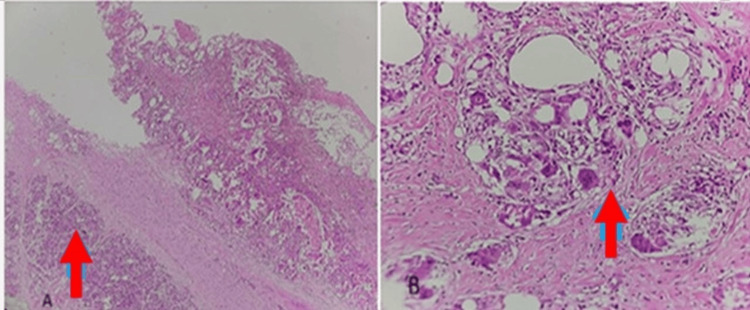
Histology slides stained with hematoxylin and eosin. (A) Low-power view (10x) showing pancreatic tissue adjacent to a cyst with extensive fat necrosis and multinucleated giant cells (arrow pointing to pancreatic acini). (B) High-power view (40x) showing areas of fat necrosis and multinucleated giant cells (arrow pointing to giant cells).

Histopathology of the excised specimen showed a cystic soft tissue fragment weighing 148 grams and measuring 13.5 x 7 cm, with a wall thickness of 0.1 to 1.5 cm. The external surface was grey-brown in color, with areas of greying and black discoloration. Microscopy revealed pancreatic tissue with an adjacent cystic lesion containing large areas of fat necrosis, foreign body giant cells surrounding golden-yellow pigment, and acute-on-chronic inflammatory granulation tissue with hemorrhagic exudate. Adjacent pancreatic tissue showed features of chronic pancreatitis, while the adrenal tissue appeared unremarkable (Figure 2). No evidence of malignancy was noted.

**Figure 3:**
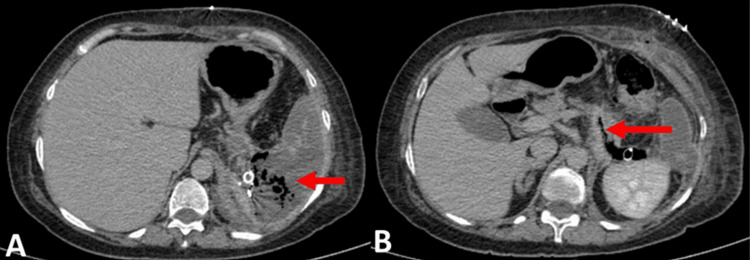
Contrast-enhanced computed tomography (coronal section). A. Spleen diffusely hypodense and non-enhancing. B. Few air foci through the parenchyma communicating with the collection.

On postoperative day 10, the patient presented to the outpatient department with abdominal pain and vomiting. Examination revealed a soft abdomen with bowel sounds present. The drain bag was distended with gas and feculent discharge, raising suspicion of a more severe complication. A CECT of the abdomen was performed, which showed the distal pancreas to be bulky with non-enhancing areas, and few linear air foci communicating with the distal pancreas, suggesting pancreatic duct injury and devascularization of the spleen (Figure 3). Emergency exploratory laparotomy was planned.

Emergency management included exploratory laparotomy with splenectomy, distal pancreatectomy, resection of the splenic flexure, transverse colostomy, and formation of a distal mucus fistula. Postoperative recovery was monitored closely, and the findings were consistent with a diagnosis of left adrenal myelolipoma complicated by surrounding organ involvement.

## Discussion

This case underscores the diagnostic challenges encountered when a pancreatic pseudocyst mimics an adrenal myelolipoma. Although rare, pancreatic pseudocysts can occasionally be misdiagnosed as adrenal myelolipomas due to overlapping radiological features, with such diagnostic errors reported in up to 5-10% of cases involving retroperitoneal fat-containing lesions [6].While myelolipomas are typically benign and often discovered incidentally, their size and location can create diagnostic dilemmas, especially when they coincide with other pathologies. Our patient had back pain, fever, vomiting, and fatigue, which were suggestive of an adrenal mass. Yet, later complications such as foul-smelling discharge and fistula development of a pancreatic fistula suggested otherwise. The histopathological examination showed a cystic lesion with fat cell apoptosis, chronic inflammation, and chronic pancreatitis, making the diagnosis of pancreatic pseudocyst possible.

Pancreatic pseudocysts can occasionally mimic retroperitoneal masses such as adrenal myelolipomas, leading to diagnostic confusion on initial imaging and clinical evaluation [4,5]. In this case, overlapping symptomatology and early radiologic interpretation favored a myelolipoma, thereby obscuring the underlying diagnosis of a pancreatic pseudocyst. This highlights the importance of maintaining a broad differential diagnosis, especially when clinical presentation and imaging findings are incongruent. The use of additional imaging modalities, such as magnetic resonance imaging or endoscopic ultrasound, may have provided further characterization of the lesion and facilitated an earlier and more accurate diagnosis. Furthermore, the diagnostic delay may have been compounded by the patient’s history of type II diabetes mellitus, which can obscure or modify typical symptom patterns [6-8].

The laparoscopic method had to be converted to open surgery owing to an increase in scar tissue. This reciprocates the difficulties in operating on large retroperitoneal masses whether they are myelolipomas or pseudocysts [9-12]. The postoperative complications led to additional surgeries. This case underscores the need for thorough surgical planning and teamwork when managing complicated retroperitoneal problems.

The presence of chronic pancreatitis adjacent to the cystic lesion raises the question of which occurred first. It may be possible that the pseudocyst was the primary issue, thereby spreading the inflammation to the nearby pancreatic tissue. The type II diabetes mellitus in the patient may have caused pancreatitis, thereby resulting in pseudocyst formation [3].

The increased level of amylase in the drain in the postoperative period confirmed the diagnosis of pancreatic fistula, which is a known complication of pancreatic surgeries. Managing such procedures requires a team effort, often demanding expertise from surgical gastroenterology.

This case emphasizes the need for detailed diagnostic evaluation and a strong suspicion when examining patients with retroperitoneal masses. The chance of a pancreatic pseudocyst imitating an adrenal myelolipoma should be taken into consideration in such unusual presentations.

Take-home message

The paranephric fat abutting the tail of the pancreas may resemble a mass originating from the suprarenal gland. Lifting the pancreas tissue does not happen in an adrenal mass. Adrenal myelolipoma dissection will not be difficult, and, therefore, conversion to open will be less likely in such cases. Conversion to open should ring an alarm. Prompt diagnosis, and immediate and appropriate treatment for pancreatic fistula are life-saving.

## Conclusions

In conclusion, the noticeable mass in the left upper abdomen, with its unclear and vague edges, was unusual for an adrenal issue. Surgical observations of strong adhesions to the spleen, the tail of the pancreas, and the upper part of the kidney, along with considerable bleeding, further strayed from the typical traits of an adrenal mass. Preoperative CT showing displacement and lifting of the pancreas raised additional concerns, as such findings are unusual for a lesion confined to the adrenal gland. Retrospective analysis of the case highlights the importance of considering alternative diagnoses in complex retroperitoneal masses, emphasizing the need for careful correlation of clinical, radiological, and intraoperative findings to avoid potential diagnostic and therapeutic pitfalls.
